# A Transformative Subfield in Rehabilitation Science at the Nexus of New Technologies, Aging, and Disability

**DOI:** 10.3389/fpsyg.2012.00340

**Published:** 2012-09-21

**Authors:** Carolee J. Winstein, Philip S. Requejo, Elizabeth M. Zelinski, Sara J. Mulroy, Eileen M. Crimmins

**Affiliations:** ^1^Division Biokinesiology and Physical Therapy at the Herman Ostrow School of Dentistry, University of Southern CaliforniaLos Angeles, CA, USA; ^2^Department of Neurology, Keck School of Medicine, University of Southern CaliforniaLos Angeles, CA, USA; ^3^Rancho Los Amigos National Rehabilitation CenterDowney, CA, USA; ^4^Leonard Davis School of Gerontology, University of Southern CaliforniaLos Angeles, CA, USA

**Keywords:** aging, disability, technology, eHealth, rehabilitation, chronic care model, quality of life, interactive media

## Abstract

We argue that a silo research and training approach is no longer sufficient to provide real solutions to the complex humanitarian, social, and financial problems brought about by global trends in aging and the increased prevalence of multiple chronic conditions that limit independence and activities of daily living. This perspective highlights the opportunities for collaborative research and training in a new multidisciplinary science of rehabilitation enabled by growing knowledge and information along scientifically and clinically meaningful lines. The recent proliferation of eHealth technologies offers opportunities for development of low-cost, simple, interactive media prevention, health maintenance, and continued functional recovery programs using a chronic care model designed to promote engagement and participation. With two examples – long-term disability consequential to (1) hip fracture and (2) manual wheelchair use – we outline the developing science for a collaborative and transformative nexus team capable of accelerating an understanding of ways to restore independence and improve quality of life, in the long-term. We conclude with a set of recommendations for the design of interactive media systems to both increase acceptability and stimulate research.

## Introduction

Traditional rehabilitation research and training is constrained by a predominantly specialist approach that runs counter to the complex societal needs posed by the global aging crisis (Rae et al., 2010) facing today’s world health care systems. Specifically, as the USA’s 76 million baby boomers begin to retire, our health care system faces a demand unprecedented in the history of the nation. Millions of new patients, many with multiple age-related chronic conditions will contribute to unsustainably soaring costs that demand entirely new ways of delivering care, let alone conducting research and training the next generation of scientists and clinicians (Conway and Clancy, 2010; U.S. Department of Health and Human Services, 2010; Institute of Medicine, 2012). Relevant to rehabilitation science and technology, there are a number of trends in society that are exhibiting exponential growth including: computer processing power, data storage, bandwidth, nanotechnology, and advances in robotics and brain scanning technology. An interesting characteristic of technology is that when it grows exponentially, it can also get better, faster, cheaper, and smaller, and as it does, it spreads out into society. A recent example of this rapid diffusion process is how quickly people began using the iPad device^1^. Interestingly, marketers who conducted focus groups during the iPad development phase found that most consumers could not see any use for such a device; as such, the marketers did not predict this rapid public adoption.

For these reasons, the time has come for the emergence of a transformative subfield in rehabilitation science at the nexus of new interactive media technologies, aging, and disability research. This interdisciplinary subfield should be capable of building capacity by leveraging new ideas and technologies to not only increase, but to accelerate our understanding of ways to restore independence and improve the quality of life for the growing population of older adults, people with disabilities, and those aging with disabilities (National Research Council, 2011). This is a necessary first step to modernize rehabilitation practice and to outline important aspects of prevention, health maintenance, and continued functional recovery that can inform critical decisions in healthcare management and reform.

There is considerable recognition that aging and disability are two arenas that are increasingly viewed as a national and international problem requiring effective, practical, and scalable solutions (Rae et al., 2010; Seeman et al., 2010; Wyke, 2011; Steel et al., 2012). The recent proliferation of low-cost technologies including, for example, mobile health monitoring devices (e.g., Lumoback^2^), interactive uses for the Kinect technology in rehabilitation, in an operating room in Spain, and helping children with autism spectrum disorder (e.g., Microsoft Kinect system^3^), and simple emergency notification devices (e.g., Phillips Lifeline^®^^4^), offer a glimpse of what is already available both inside and outside of formal healthcare services. The technological advances that have enabled these developments allow tremendous opportunities and new options for service providers, researchers, advocates, practitioners, and an increasingly larger number of users. Indeed, the recent 33rd Centers for Disease Control (CDC) report on health in the US (National Center for Health Statistics, 2010) acknowledged that technology continues to transform the medical care system, but the report is limited in its inclusion of the newer low-cost interactive technologies that have only recently become available. This example highlights the need to jump the curve; that is as the global futurist; Uldrich (2008) says “Do not stay ahead of the technology curve….Jump over it.” Collaborative research and training programs at the nexus are well positioned to leverage the growth in computing technology to expand beyond the traditional rubric of “assistive technologies” in ways that have not yet been imagined. The scientific and social impact of overcoming the traditional specialist approach and joining together scientists and practitioners from engineering, computer science, gerontology, and the rehabilitation professions around real problems is likely to be transformative as it has the potential to accelerate advances that address important societal needs and are essential for translational research (Woolf, 2008).

Indeed, there are several new joint training programs in the USA where medical students and biomedical engineering students co-train and learn together about each other’s science and practice (e.g., Health Technology Education at USC; Health Sciences and Technology at Harvard). These innovative and interdisciplinary training programs aim to train a new generation of leaders in health care who are conversant in the language of multiple disciplines. There is an important difference between these joint medical and engineering programs and the engineering/aging/disability nexus collaboration outlined here. Medical/engineering programs are primarily based on an acute care model with a focus on finding solutions to immediate medical or biological problems while the engineering/aging/disability nexus is primarily based on a chronic care model with a focus on finding solutions to longer-term biological, psychological, and social problems. Naturally, this new subfield of rehabilitation science is focused on *prevention, health maintenance and continued functional recovery for those aging into or with disabilities*. In the USA, the National Institute for Disability and Rehabilitation Research (NIDRR) showed remarkable vision in formulating a research agenda to develop Rehabilitation Engineering Research Centers (RERC) of excellence whose goals were, “to conduct research, demonstration, and training activities regarding rehabilitation technology in order to enhance opportunities for meeting the needs of, and addressing the barriers confronted by, individuals with disabilities in all aspects of their lives” (NIDRR, 1998). One of these centers is specifically focused on the growing population of those aging into and with disabilities. These programs have generally been successful, but the recent National Research Council review made several recommendations to increase the chances of producing the highest quality outputs (National Research Council, 2011). The review called for the formation of a standing Rehabilitation Research Advisory Council to advise the director of the agency on research priorities and the development of the agency’s Long-Range Plans. This recommendation will be important for assuring that the Long-Range Plans including “rehabilitation technology” are aligned with the most current science and developments that most certainly will include the growing field of interactive media technologies.

To date, while individual research groups in biomedical engineering including interactive media (e.g., Lange et al., 2010; Profitt and Lange, 2012), assistive technologies (e.g., Helal et al., 2008), rehabilitation science (e.g., Souza et al., 2010; Mulroy et al., 2011a), and aging and disability studies (e.g., Iezzoni and Freedman, 2008; Mann, 2008) are actively advancing the science in important and incremental ways, there is still limited cross-talk between these groups (see Putnam, 2007; Washko et al., 2012, for a few recent exceptions). As such there remain significant barriers to assembling teams of researchers with a common vision who are conversant in the language of multiple disciplines.

## Why Now? Compelling Societal Needs and Trends

The three most compelling reasons to target the aging baby boomers and those aging with and into disability are: (1) this is the fastest growing age group in the world (Rae et al., 2010), (2) healthcare costs are highest among the older group; the elderly (age 65 and over) made up around 13% of the USA population in 2002, but they consumed 36% of total USA personal health care expenses. The average health care expense in 2002 was $11,089 per year for a person 65 or older but only $3,352 per year for a working-age person (ages 19–64; Keehan et al., 2004), and (3) clinically, this group has a high rate of chronic conditions and functional limitations resulting in a high health care utilization. Longer life spans are generally considered desirable, particularly when healthy years of life are increased. But with an aging population and longer life expectancy come an increasing prevalence of chronic diseases and conditions associated with aging (Kemp and Mosqueda, 2004; Crimmins and Beltran-Sanchez, 2011), including hypertension, diabetes, end-stage renal disease, and certain types of cancer, as well as Alzheimer’s disease and other dementias (National Center for Health Statistics, 2010) that can significantly reduce quality of life. According to data from the USA Census Bureau, approximately 19% of Americans have a disability involving an activity limitation, and the incidence of disability increases with age (Brault, 2012). Although disability is more prevalent among males at younger ages, it is more prevalent among females at older ages. On the basis of data from the National Health Interview Survey, 18% of working-age adults have a disability, and this increases to 54% among older adults. Although disability is often considered a progressive process, it is also a dynamic one, and individuals often move in and out of disability states (Crimmins et al., 1994). More importantly, we now know that in many cases, the disability state can be modified through targeted simple, low-cost prevention, health maintenance, or continued functional recovery programs that consider psychological, physiological, and environmental factors. In the following sections, we outline two examples (i.e., hip fracture; chronic disability in wheelchair users), first by defining the complexity of the problem, and then highlighting how an engineering/aging/disability team might develop and use technology to support the delivery of effective long-term care; that which concurrently addresses the biological, psychological and social aspects inherent in the disability and which aims to restore independence and improve quality of life.

## Long-Term Disability after Hip Fracture

Of the 300,000 Americans 65 years or older who fracture a hip each year, 20–30% will die within 12 months, and “many more will experience significant functional loss,” according to a 2009 study published in the *Journal of the American Medical Association* (Sennerby et al., 2009). The statistics are quite startling: a year after fracturing a hip, 90% of those who needed no assistance climbing stairs before the fracture will not be able to climb five stairs; 66% will not be able to get on or off a toilet without help; 50% will not be able to raise themselves from a chair; 31% will not be able to get out of bed unassisted; and 20% will not be able to put on a pair of pants. But what makes a hip fracture so deadly and so debilitating? And how can a seemingly healthy person experience such a dramatic decline after suffering what is essentially just a broken bone? An understanding of the complexity of issues is gained by unraveling the multiple sub-threshold conditions such as subtle cognitive deficits, decreasing bone density and muscle weakness that may not have reached a critical threshold, but together amplify the decline. As people age, they also experience co-morbidity or multiple ailments at the same time as indicated in the earlier discussion of disability. In fact, most older adults have at least one chronic condition such as diabetes or heart problems and many have more than one when they fall and break a hip. It is now understood that a hip fracture is an insult to multiple systems, and can result in many metabolic and cognitive changes to an already challenged system (Alexander and Hausdorff, 2008). There is also evidence of reverse causality, for example when a cognitive deficit such as dual tasking drains attentional resources, this can compromise balance and can lead to a fall that causes a hip fracture (Ojha et al., 2009). Basically, and in the context of multiple systems, the answers have less to do with the break itself than with the physiological and psychological reaction to the break, and not just in the hours immediately following but also in the days, weeks, and months post-injury. As one ages and bones weaken, a fall that children or grandchildren might walk away from could result in a hospitalization and major surgery. That surgery carries risks that are greater when one is older and so does the immobility caused by a broken hip. When an older person is bedridden and hospitalized even for a few days, the odds of infection, inflammatory processes, atrophy of bone, muscle, brain, central nervous function, and pneumonia increase dramatically.

Consistent with a medical model perspective, most of the research has focused on the acute treatment phase. As such, there are some acute strategies that can improve the odds. For example a recent study found that the risk of death from a hip fracture declined by 19% when surgery was performed within 3 days of the break (Simunovic et al., 2010). At the point when standard services end, after about 3 months, the real work begins – a point lost on many who have experienced a hip fracture. Professor Rebecca Craik, describes the usual expectations and the failing medical system when it comes to rehabilitation after a hip fracture. “We like to say it is only a broken bone and bones heal. But when you are an older adult, the bar is frequently set too low: It is good enough to just be home. It is time in your life to rest. Patients and their caregivers often do not push to get where they were before the surgery” (Slear, 2011). Part of the problem is that after a few months, just as the patient gains the ability to endure the intense rehabilitation that will restore pre-fracture mobility, the infrastructure for healthcare evaporates.

## Interactive Media Technology to Restore and Maintain Function after Hip Fracture

Low-cost, innovative technologies can be utilized to facilitate at least four effective strategies to enable recovery, restore independence, and improve quality of life: (1) *Daily exercise* – in particular, weight-bearing activity can stimulate bone growth (i.e., slowing bone loss with age) and healing (i.e., in the case of a hip fracture) – simple sensors in the shoe; smart phone apps (California Healthcare Foundation, 2010), or commercially available activity monitors such as the FitBit^5^ can be used to track the amount of weight-bearing activity that is done, provide feedback and even daily target levels for a gradual progression that approaches age-appropriate activity levels. There is evidence that a virtual reality exposure therapy (VRET) program can be effective for the reduction of fear of falling and balance rehabilitation training of elder adults with hip fracture history (Giotakos et al., 2007); (2) *Proper diet* – There are even interactive media programs and smart phone apps to monitor diet and in particular, protein intake, calcium and vitamin D. Simple sensors in pill dispenser packaging can be used to monitor compliance and trigger a text message or some smart phone app reminder if the pill has not been taken that day, and encourage the individual to take in appropriate levels of nutrients such as protein everyday; (3) *Socialize* – more social interaction leads to a stronger desire to get out of the house, which provides more impetus for better self-care. It is about staying engaged in life and maintaining a desirable quality of life. Here is where technology that enables social communication – the Internet and social media could play an important role (Madden, 2010; Smith, 2010; Valente, 2010). Interestingly, blogging and online health discussions are two activities that are more frequent among people living with chronic disease. Having a chronic disease significantly increases an Internet user’s likelihood of saying they work on a blog or contribute to an online discussion, a listserv, or other online group forum that helps people with personal issues or health problems (Fox and Purcell, 2010). Chronic disease among Internet users is also associated with a greater likelihood of accessing user-generated health content such as blog posts, hospital reviews, doctor reviews, and pod casts (Smith, 2010). Finally, (4) *Persistence* – health care provided therapy requires a significant commitment, but maintaining an active life style is most important in the months and years following the supervised physical therapy. Both self-confidence in balance and gait activities and a more general feeling of competence are shattered after an unexpected hip fracture or disabling neurological condition like stroke or traumatic head injury and even a “simple” knee replacement. The feedback about success and accomplishments provided by smart interactive technology can be important to help build up self-confidence in dynamic balance and a feeling of competence – one of the three basic psychological needs that has been shown to be very important, especially after a breach of one’s confidence occurs (Bandura, 1997; Lewthwaite and Wulf, 2010a,b).

## Long-Term Disability in Wheelchair Users

Approximately 12,000 new survivors of spinal cord injury (SCI) are added each year to the total population of approximately 300,000 persons now living with SCI in the United States (National Spinal Cord Injury Statistical Center, 2011). Prevalence of SCI worldwide has been estimated to range from 223 to 755 per one million inhabitants (Wyndaele and Wyndaele, 2006). Both improved life expectancy and increasing age at injury have resulted in a population that is increasingly experiencing the impact of aging with a disability (Kemp and Thompson, 2002). One of the most common secondary complaints in the SCI population is shoulder joint pain, which has been attributed to the high demand on the upper limbs during upper extremity weight-bearing activities (Bayley et al., 1987; Dalyan et al., 1999). The prevalence of shoulder joint pain after SCI is greater than in the non-disabled population at every age and increases steadily with time after injury impacting a full 70% of individuals at 20 years post SCI (Sie et al., 1992). Because individuals who use a manual wheelchair (WC) are dependent on their upper extremities for mobility and daily activities, shoulder dysfunction and pain presents an additional loss of independence and decreased quality of life (Gutierrez et al., 2007). This clinical problem was identified in the scientific literature in the 1980s and 1990s (Silfverskiold and Waters, 1991; Sie et al., 1992; Pentland and Twomey, 1994) and unfortunately, its prevalence remains high today (Gironda et al., 2004; McCasland et al., 2006; Jain et al., 2010). Moreover, it is largely an untreated problem (Alm et al., 2008; Brose et al., 2008); perhaps untreated because (1) it does not fall within the domain of the acute care model, and (2) the paucity of experienced engineering/aging/disability teams prepared to find an effective solution.

The most common pathology documented in individuals with paraplegia and shoulder pain was chronic subacromial impingement syndrome; with 65% of individuals displaying evidence of rotator cuff tear (Escobedo et al., 1997). Moreover, in a recent study of wheelchair athletes, supraspinatus tendinopathy and impingement was detected in nearly all participants (Brose et al., 2008). The chronic impingement and shoulder joint pain that occur after SCI likely have multi-faceted causes. The activities that provoke the highest pain responses for manual WC users are also those that are repetitive and generate high shoulder forces including transfers, entering/leaving a car, loading a WC into a car, ascending ramps, heavy lifting with arms and outdoor wheeling (Curtis et al., 1999; Gironda et al., 2004; Samuelsson et al., 2004; Alm et al., 2008; Fliess-Douer et al., 2012).

To address this clinical problem, we recently evaluated the efficacy of a non-surgical rehabilitative approach in the first randomized control trial for treating shoulder pain in persons with SCI, “Strengthening and Optimal Movements for Painful Shoulders in persons with SCI (STOMPS).” We demonstrated a marked reduction in shoulder pain after a 12-week home-based shoulder stretching and muscle strengthening exercise program that was combined with instruction to optimize the performance technique of pain-inducing tasks for each participant (Mulroy et al., 2011a). Shoulder pain was documented in our clinical trial with the Wheelchair User’s Shoulder Pain Index (WUSPI; Curtis et al., 1995). In the experimental group WUSPI scores were reduced from 51 (out of a maximal possible score of 150) to 15 after the 12-week intervention while the scores of the control group were unchanged at 45 (Mulroy et al., 2011a). Moreover, after this relatively brief intervention, participants in the experimental group reported statistically significant improvements in health-related and overall subjective quality of life compared to the control group. A mediation analysis of these results identified that the intervention-induced reduction in shoulder pain was the direct cause (mediator) of the improvements in participation and overall quality of life seen in our participants (Mulroy et al., 2011b). Our results were particularly impressive as the average duration of shoulder pain in our study cohort was 5 years.

While the overall response to the intervention was strong, in our trial as well as in the prior studies of shoulder exercises in SCI, pain reduction in the intervention group participants was not complete for all persons (Curtis et al., 1999; Nawoczenski et al., 2006; Mulroy et al., 2011a). Ninety percent of subjects in the intervention group in our trial had a substantial reduction in shoulder pain level, yet 30% of subjects still had WUSPI scores greater than 25 after the intervention (Mulroy et al., 2011a). Ideally, a preventative program to preserve shoulder function would be employed to maintain independence and quality of life for all persons after SCI. Our current approach is to develop technological solutions for translating our simple, home-based intervention that was successful at reducing chronic pain into an effective preventative program. Despite the fact that prevalence of shoulder pain increases with time post-injury, response to the intervention was not related to the participant’s age, duration of spinal injury, or duration of shoulder pain, which should be encouraging to clinicians and patients in that the program can be effective even for older individuals or those with longstanding shoulder pain.

## Interactive Media Technology to Preserve Shoulder Function in Chronic Wheelchair Users

Technological solutions for prevention of shoulder pain in individuals with SCI present the opportunity to proactively address potential barriers to exercise in this population. A recent survey of persons with SCI regarding barriers to exercise identified three primary areas: (1) intrinsic (e.g., lack of motivation, energy, or interest), (2) resources (e.g., cost of an exercise program, not knowing where to exercise), and (3) environmental (e.g., accessibility of facilities and knowledgeable instructors; Scelza et al., 2005). Our engineering/aging/disability team is developing a virtual reality shoulder game designed to incorporate the STOMPS protocol into a game-based tool that would counter each of the three identified barriers to exercise.

The release of the low-cost Microsoft Kinect camera and the widespread accessibility of game programming (e.g., Unity^6^, FAAST^7^ software allowed our multidisciplinary team to accelerate the development of the STOMPS exercise game for wheelchair users. The Kinect system does not require the user to hold an interface device or move on a pad as the source of interaction within the game. Instead, the user’s upper extremity is the game controller operating in 3D space and multiple users can be tracked in this fashion for both cooperative and competitive gaming activities. We have been creating different game scenarios within the game-based shoulder exercise system. The system is composed of a frame that accommodates a standard wheelchair; and is outfitted with elastic resistance bands (Theraband^®^) that are sensorized with a 3D tracker (Gametrak), a Microsoft Kinect, a laptop and a large screen. The four STOMPS exercises are presented separately and the player or clinician can make choices about the number of sets and repetitions. Intrinsic motivation and interest would be facilitated by providing choices and creating an engaging game scenario to accompany the exercises (Annesi and Mazas, 1997; Mestre et al., 2011; Chiviacowsky et al., 2012).

The game scenarios incorporate gaming features (movement boundaries and goals) that encourage the player to perform the shoulder strengthening exercises with appropriate body position and control. We implemented a feedback system that provides the player with feedback on their performance in real time and to ensure the exercises are performed correctly. This combination of features would help to bring knowledgeable instruction to the individual. The long-term goal is to develop an inexpensive, home-based virtual reality shoulder exercise system.

## Technology-Promoting Healthy Behavior

### New technologies and behavior change within a chronic care model

Indeed, the acute medical management is only a small part of the strategy to address the long-term consequences that such multi-faceted and complex conditions which typically develop after hip fracture, stroke, SCI, traumatic head injury, Parkinson’s disease, for examples. Each of these conditions may present initially with acute medical needs, but the best-known framework about care for people with long-term conditions and disabilities is the Chronic Care Model (Wagner et al., 2001; Bodenheimer et al., 2002) that has gained international visibility in recent years. The Chronic Care Model can more easily embrace low-cost and often-simple therapeutic strategies to restore function, monitor health, and prevent future decline while engaging in a healthier lifestyle focused on overall improved quality of life (Wang and Liu, 2009). As Thomas Gill recently noted, “because of decades-long trends toward shorter hospital stays, patients often are discharged to sub acute facilities earlier than in the past, despite underlying conditions. Thus, when they return home, patients often are recovering from underlying medical conditions and from declines or disabilities that led to the hospitalization. Rehabilitation in older persons therefore occurs primarily in the home, most commonly through a visiting nurse service supported by Medicare” (National Institute on Aging, 2010).

The two examples described earlier provide an explicit illustration of how the output of research at the engineering/aging/disability nexus is not only complementary, but also completely consistent with the goals of the chronic model of care where not only the biological, but also the psychological and social realities of the individual are of the utmost importance. One’s attitude and expectations following a hip fracture or any traumatic event, for that matter, can have a tremendous impact on the recovery and restorative processes that ensue. In addition, there is little appreciation for how modifiable a disability state can actually be (i.e., see earlier discussion). As such, expectations for recovery outside the acute phase are often limited – leading to complacency and a naïve acceptance of an inevitable decline.

For example, those living with chronic SCI seem to accept shoulder pain as an inevitable consequence of using their wheelchair for mobility. In a recent study by Brose et al. (2008) they noted, “Roughly one fourth of our subjects had shoulder pain that limited their activity and was not treated by a physician.” They further posited, “Persons with SCI learn to cope with impairments, and it is possible that clinically significant shoulder pain is under appreciated in this group.”

There is growing evidence across diverse disability groups that patient/client centered programs and strategies focusing on prevention, improved independence and quality of life by considering basic psychological needs can enable participation, change behaviors, reduce stress, modify lifestyle factors, enable a healthier lifestyle over time and revise expectations (Bandura, 1997; Hammell, 2007; Khaw et al., 2008; Kang et al., 2010; Lewthwaite and Wulf, 2010a,b; Walsh, 2011). While there are specific needs for different diagnostic groups (e.g., hip fracture, chronic SCI) that are important with respect to reducing disability, there are a few cross-cutting and common lifestyle factors that are equally important considerations for the development of research and training programs at the nexus. Walsh (2011) argues that differences in four lifestyle factors: that of smoking, physical activity, alcohol intake, and diet exert a major impact on mortality and points to a critical study by Khaw et al. (2008) showing that even a small difference in lifestyle can make a major difference in health status. These lifestyle factors can be incorporated into the design of interactive media tools to encourage self-management strategies focused on prevention, improved independence and quality of life.

Some recent examples of interactive media tools that show promise for those aging into or with disabilities include: traumatic head injury and particularly those with concomitant PTSD (e.g., virtual reality exposure assessment and therapy programs to promote community re-integration (Rizzo et al., 2011, 2012); cardiac bypass surgery (e.g., diet/nutrition and exercise programs, smoking cessation; behavioral health – see Thrive Research, Inc.,^8^); cerebral vascular accident/stroke (e.g., home and community exercise and virtual reality game programs (Henderson et al., 2007; Levin, 2011); rehabilitation robotics for stroke (Wade and Winstein, 2011), Parkinson’s disease (e.g., wearable sensors to detect freezing of gait, (Bachlin et al., 2010); and Alzheimer’s disease (e.g., verbal and visual instruction technologies for daily activities (Lancioni et al., 2008, 2012).

## Recommendations to Increase Universal Acceptability and Stimulate Research

As the previous examples illustrate, and as the need for inclusion of lifestyle factors increases due to the prevalence of unhealthy behaviors such as overeating and lack of exercise (“globesity,” World Health Organization, 2008), the opportunities for new interactive media technologies such as immersive technologies (e.g., Virtual Reality), exer-gaming (e.g., using Microsoft Kinect technology), and mobile monitoring devices including smart phone apps will grow to provide low-cost and user-engaging solutions to the tremendous medical, psychological, and economic cost of those aging into and with disability. As mainstream use of the various applications and social media tools continues to soar, providers can harness this trend to improve their practices; communicating with clients does not have to end with face-to-face meetings. This new subfield of rehabilitation science is well positioned at the outset to accelerate evidence-based research to inform practice and development toward long-term behavioral changes that promote a healthy lifestyle with a focus on prevention and wellness in those aging with and into disability (Lange et al., 2010).

The following recommendations are provided to increase acceptability and stimulate research in this new nexus subfield of rehabilitation science. New interactive media technologies should be designed: (1) to be “smart” – this means that the technology must address the clinical problem and be able to detect the critical features that predict certain behaviors. Note that this requirement will stimulate important research at the nexus of new technologies, aging, and disability. (2) To be personalized to the individual who will use it – this might be accomplished through some learning algorithms where the device learns the specific behaviors and preferences of its user^9^, (3) to be simple to use, easy to see (e.g., read the screen)- screens and feedback should be intuitive in nature, this means that it must be accessible (National Research Council, 2011) and embody the principles of Universal Designs (Center for Universal Design, 1997), and, (4) with age-sensitive, racial, ethnic, and cultural specificity to the individual user and thereby embrace the global nature of this growing challenge and opportunity (Rae et al., 2010; National Research Council, 2011).

## Conclusion

This perspective highlights the opportunities for evidence-based research and training in a new multidisciplinary science of rehabilitation enabled by growing knowledge and information at the nexus of new technologies – specifically interactive media technologies – aging, and disability research. The recent proliferation of eHealth technologies that are rapidly diffusing into the global society, offer new opportunities for the development of low-cost, simple, patient/client centered, interactive media prevention, health maintenance, and functional recovery programs for those aging into and with chronic disabilities. We suggest that research emergent from this new multidisciplinary subfield of rehabilitation is more likely to accelerate advances in rehabilitation science and practice than any of the specialty areas working alone. We use two clinical examples of disability, one consequential to a late life hip fracture that likely is accompanied by one or more chronic conditions (e.g., heart disease, diabetes, arthritis), and the other an over-use shoulder pain syndrome in chronic wheelchair users to illustrate the important role for this new nexus subfield. Each clinical condition, though different by diagnostic group, shares an important and meaningful outcome, that which threatens function and independence and severely reduces the quality of life. We outline the developing rehabilitation science for both of these conditions including that associated with complex psychological, physiological, and environmental factors. We suggest ways that a transformative multidisciplinary team who is conversant in the language and research methods of the nexus subfield could use that knowledge in the development of interactive media technology designed to restore independence and improve quality of life, in the long-term. We conclude with a set of recommendations for the design of interactive media systems to both increase acceptability and stimulate important future research.

## Conflict of Interest Statement

The authors declare that the research was conducted in the absence of any commercial or financial relationships that could be construed as a potential conflict of interest.

## Footnotes

^1^http://diffusedipad.wordpress.com/2011/03/15/diffusion-of-innovation/

^2^http://www.lumoback.com/

^3^http://www.xbox.com/en-US/Kinect/Kinect-Effect

^4^http://www.lifelinesys.com/content/home

^5^www.fitbit.com

^6^http://unity3d.com/

^7^http://projects.ict.usc.edu/mxr/faast

^8^http://www.thriveresearch.com/

^9^http://www.careinnovations.com/independent-living
